# Oral membrane-biomimetic nanoparticles for enhanced endocytosis and regulation of tumor-associated macrophage

**DOI:** 10.1186/s12951-023-01949-5

**Published:** 2023-07-04

**Authors:** Xiaoyan Gu, Rongguang Zhang, Yingwei Sun, Xinyi Ai, Yu Wang, Yaqi Lyu, Xiaoyu Wang, Yihan Wu, Zhi Wang, Nianping Feng, Ying Liu

**Affiliations:** 1 School of Pharmacy, Shanghai University of Traditional Chinese Medicine, 1200 Cailun Road, Zhangjiang Hi-Tech Park, Pudong New District, Shanghai, 201203 P R China; 2 Experiment Center for Science and Technology, Shanghai University of Traditional Chinese Medicine, Shanghai, 201203 China

**Keywords:** Membrane-biomimetic, Enterocyte uptake, Overcoming intestinal barriers, Regulation of tumor-associated macrophage

## Abstract

Enterocyte uptake with high binding efficiency and minor endogenous interference remains a challenge in oral nanocarrier delivery. Enterocyte membrane-biomimetic lipids may universally cooperate with endogenous phosphatidyl choline via a biorthogonal group. In this study, we developed a sophorolipid-associated membrane-biomimetic choline phosphate-poly(lactic-*co*-glycolic) acid hybrid nanoparticle (SDPN). Aided by physical stability in the gastrointestinal tract and rapid mucus diffusion provided by association with sophorolipid, these nanoparticles show improved endocytosis, driven by dipalmitoyl choline phosphate-phosphatidyl choline interaction as well as its optimized membrane fluidity and rigidity. Luteolin- and silibinin-co-loaded with SDPN alleviated breast cancer metastasis in 4T1 tumor-bearing mice by regulating the conversion of tumor-associated M2 macrophages into the M1 phenotype and reducing the proportion of the M2-phenotype through co-action on STAT3 and HIF-1α. In addition, SDPN reduces angiogenesis and regulates the matrix barrier in the tumor microenvironment. In conclusion, this membrane-biomimetic strategy is promising for improving the enterocyte uptake of oral SDPN and shows potential to alleviate breast cancer metastasis.

**Supplementary Information:**

The online version contains supplementary material available at 10.1186/s12951-023-01949-5.

## Background

In recent years, various ligand modifications have emerged to facilitate oral nanocarrier uptake or transport through the intestinal epithelium for systemic delivery of therapeutic agents [1]. However, the limited binding orientation, high selectivity, and/or limited density of receptors might reduce ligand-receptor interaction efficiency. In addition, the orientation of the ligand must be controlled to minimize endogenous interference, for example by proteins. Therefore, providing alternatives to ligand-receptor modification and the means for improving the binding efficiency and uptake of oral nanoparticles remains a challenge. Although lipid-polymer nanoparticles (LPN) with a lipid shell and a polymeric core are attractive in oral delivery, LPN with limited biorthogonal groups and a zwitterionic structure generally have limited cellular uptake capacity, which precludes their clinical application. The cell membrane of enterocytes is mainly composed of phospholipids containing phosphatidyl choline (PC) as the head group. PC has the capacity for strong interaction with substances having a mirror-image structure [2]. Inspired by the structure of PC located on cell membranes, a biomimetic zwitterionic lipid choline phosphate (CP) was synthesized with PC-reversed head groups conjugated with a biorthogonal pendant group [2]. The structure of CP imparts its biorthogonal function, allowing easy cooperation with PC. The CP-PC interaction based on supramolecular ionic pair binding has demonstrated unique advantages that improve cellular uptake [3]. Furthermore, CP has shown protein adsorption resistance and simultaneous cell adhesion [4]. To date, CP has shown outstanding performance in tissue energy and parenteral drug delivery [5]. However, the application of CP in oral drug delivery has been precluded due to the complexity of gastrointestinal conditions and the mucus barrier.

Sophorolipids, biosurfactants produced by microorganism fermentation, are resistant to salt concentration and/or pH variation encountered in gastrointestinal tract conditions [6]. In addition, the self-assembled acidic sophorolipids may detach from the coated nanoparticles due to the binding interaction between sophorolipids and mucin during their transfer in mucus [7]. Therefore, we hypothesized that sophorolipids might exert a protective effect on CP prior to enterocyte availability.

The enhanced delivery efficiency of oral nanoparticles provides opportunities for the treatment of chronic and aggressive disease; for example, silibinin loaded or co-loaded with other therapeutic agents in oral nanoparticles has exerted regulatory effects on angiogenesis and epithelial-mesenchymal transition (EMT) [8, 9]. Due to the complex factors involved in metastasis, intervention strategies based on the accomplices and allies of tumor cell metastasis have emerged in recent years. Tumor-associated macrophages (TAM) exert a profound influence on metastasis promotion and therapy resistance [10] in breast cancer due to their unique properties and secreted cytokines or enzymes. Macrophage intervention strategies, especially the regulation of re-education of the M2 (alternatively activated) to the M1 (classically activated) phenotype, are considered valuable therapeutic tactics against metastasis. In recent years, TAM re-education has been investigated in the following three ways: by silencing M2-type macrophage-related genes such as signal transducer and activator of transcription (STAT) [11], downregulating regulatory factors that promote M2-type phenotype transformation, such as PI3Kγ inhibitors [12], and promoting M1-type phenotype transformation, such as with CD40 agonists and CD47 signal blockers [13]. STAT3 and hypoxia-inducible factor 1-α (HIF1-α) are important transcription factors involved in M2 polarization in tumor microenvironments [11, 14, 15]. The combination of STAT3- and HIF-targeting strategies is expected to maximize the regulation of macrophage polarization while reducing side effects. Another factor fueling tumor metastasis progression is the metabolic adjustments during metastatic metabolism [16]. Breast cancer cells with metastatic potential exhibit high glucose metabolic plasticity by enhancing both the glycolysis and oxidative phosphorylation pathways [17]. The activated HIF1-α in tumor tissue upregulates the activity of metabolic enzymes or proteins and enhances metabolic pathways such as glycolysis [18].

Luteolin (LU) is a natural bioflavonoid that suppresses STAT3 and HIF1-α and regulates M2-type macrophages [19]. It also inhibits c-Myc [20]. In addition, luteolin has no significant effect on the immune function of dendritic cells in the microenvironment [21]. Silibinin (SL), a bimodal Src homology-2 domain and DNA-binding domain-targeting inhibitor of STAT3 [22], has also shown regulation of EMT and inhibition of angiogenesis, which would be beneficial to comprehensively regulate the tumor microenvironment (TME) against metastasis. However, low and variable oral absorption of luteolin and silibinin hinders their clinical application. Efficient oral delivery of these agents should be considered to improve their therapeutic effect.

**Scheme 1:**
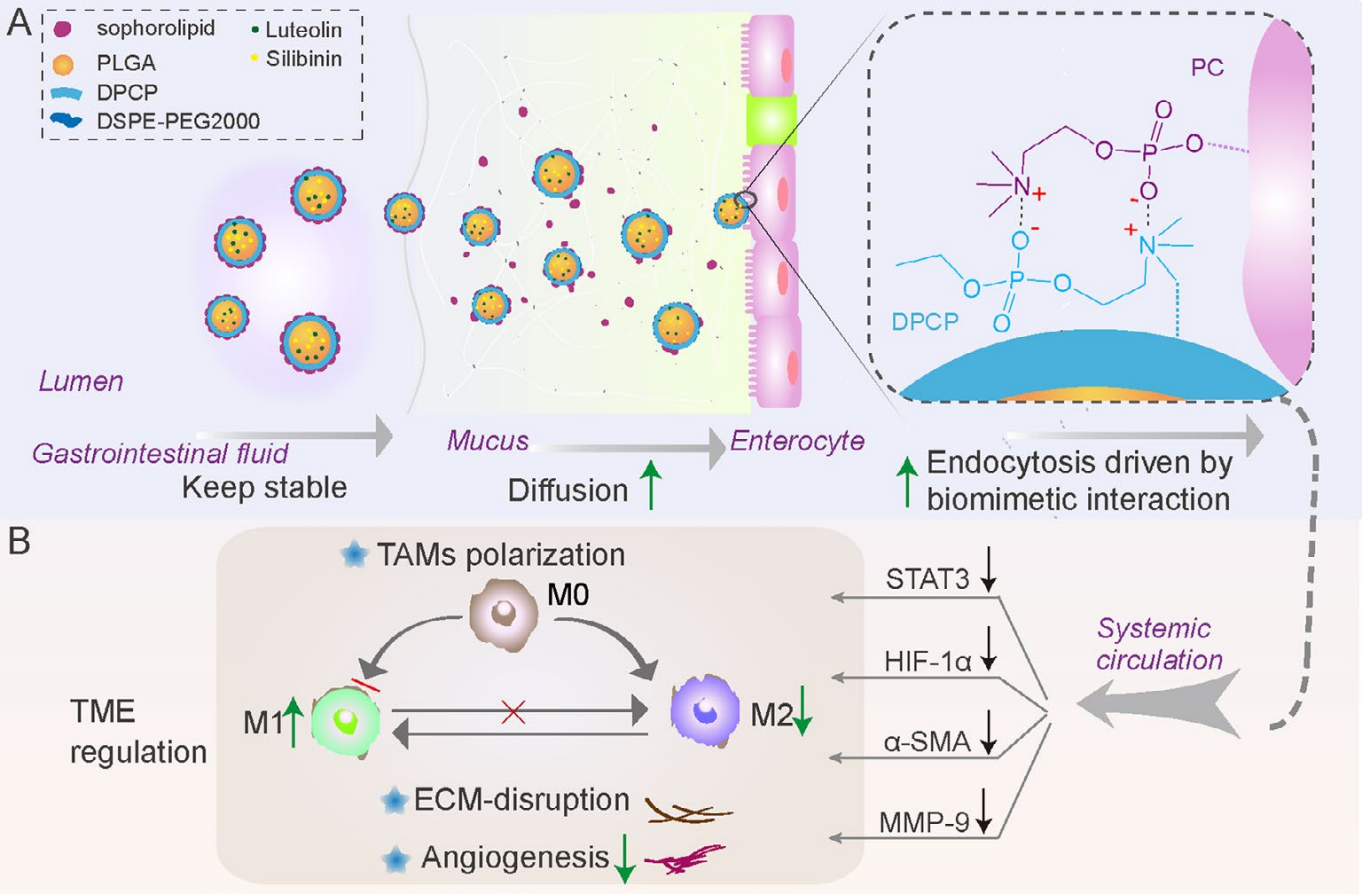
Schematic illustration of the sophorolipid-associated membrane-biomimetic choline phosphate-poly(lactic-co-glycolic) acid hybrid nanoparticle (SDPN). **(A)** Schematic illustration of physical stability in the gastrointestinal tract, rapid mucus diffusion, and improved endocytosis, driven by the dipalmitoyl choline phosphate-phosphatidyl choline interaction. **(B)** Regulation of the conversion of tumor-associated M2 macrophages into the M1 phenotype and the matrix barrier, as well as the reduction of angiogenesis in the tumor microenvironment

Here, we aim to present shell-detachable cell membrane-biomimetic oral lipid polymer hybrid nanocarriers for delivery of luteolin and silibinin (LU/SL-SDPN) (Scheme 1). As shown in Scheme 1 A, the obtained SDPN shows sophorolipid covering, which provides physical stability in simulated gastrointestinal fluid along with rapid intestinal mucus diffusion capacity. Furthermore, through its dipalmitoyl choline phosphate (DPCP) association, SDPN possesses improved enterocytic uptake driven by biomimetic CP-PC interaction, Peyer’s patch absorption, and transfer to the mesenteric vascular system. The regulation of the conversion of TAM from the M2 to M1 phenotype was proved in vitro with RAW264.7 cells and in vivo with 4T1 breast cancer tumor-bearing mice. LU/SL-SDPN lowers glycolysis and the oxidative phosphorylation metabolism capacity, which is beneficial for the alleviation of metastasis progression in terms of energy supply. The favorable action of LU/SL-SDPN on alleviating metastasis was also shown in terms of EMT process, ECM structure, and angiogenesis in 4T1 tumor-bearing mice (Scheme 1B). As opposed to the previous way of promoting endocytosis via ligand-receptor interaction, this study, inspired by the universal PC component in the enterocyte membrane, provides a new easy and more efficient way to promote endocytosis. Together with its capacity to both maintain stability in the lumen and provide rapid diffusion in mucus, this oral delivery strategy presents a new opportunity to enhance oral delivery efficiency and aid anti-metastasis treatment.

## Methods

### Materials

DSPE-PEG2000 was provided by AVT Technology Co. (Shanghai, China). Dipalmitoyl choline phosphate (DPCP) was provided by the Changchun Institute of Applied Chemistry (Changchun, China). The water-quenching NIR fluorescent probes, P2 and P4, were kindly provided by Professor Wu Wei from the School of Pharmacy, Fudan University, Key Laboratory of Smart Drug Delivery of MOE and PLA (Shanghai, China). Murine IL-4 was from Thermo Fisher Scientific (Waltham, MA, USA). Lipopolysaccharide (LPS) was from Sigma-Aldrich Co. Anti-STAT3, anti-HIF-1α, anti-c-Myc, anti-LDHA, and anti-GLUT1 antibodies were purchased from Abcam Trading Co., Ltd. (Shanghai, China). Other antibodies used in the flow cytometry assay and immunofluorescence assay were from BioLegend (San Diego, CA, USA). All other solvents were obtained from Sinopharm Chemical Reagent Co., Ltd. (Shanghai, China). All cells were purchased from Shanghai Institutes for Biological Sciences (Shanghai, China).

### Synergistic effect of luteolin with silibinin and optimum drug ratio for combination

The combined anti-tumor effect of LU and SL, as compared to each one alone, was assessed in 4T1 cells. Cell viability and IC_50_ in 4T1 cells were determined using CCK8 assays. The combination index (CI) was obtained by formula:

$$CI=a/A+b/B$$

where a and b are the doses of LU and SL used in combination, while A and B are the individual drug IC_50_ doses.

Relative mRNA expression of M1/M2 type macrophages was measured using real-time quantitative PCR (qPCR). After isolation of the total RNA from Raw264.7 cells, cDNA was reverse-transcribed using a PrimeScript RT reagent kit, as per the kit’s instructions. The cDNA mixed with the primers was subjected to qPCR (CFX Connect™ Real-Time System, BioRad, Cambridge, MA, USA) with SYBR Green Master Mix. The relative mRNA expression was obtained using GAPDH as the internal reference gene. The primer sequences of the genes are shown in Table 1.

**Table 1:** Primer sequences of the genes used for quantitative real-time polymerase chain reaction

Gene	Forward primer (5’->3’)	Reverse primer (5’->3’)
TGF-β1	CTTCAATACGTCAGACATTCGGG	GTAACGCCAGGAATTGTTGCTA
CCL2	TAAAAACCTGGATCGGAACCAAA	GCATTAGCTTCAGATTTACGGGT
TNF-α	CTGAACTTCGGGGTGATCGG	GGCTTGTCACTCGAATTTTGAGA
I NOS	GTTCTCAGCCCAACAATACAAGA	GTGGACGGGTCGATGTCAC
CD206	CTCTGTTCAGCTATTGGACGC	TGGCACTCCCAAACATAATTTGA
CD86	CTGGACTCTACGACTTCACAATG	AGTTGGCGATCACTGACAGTT
STAT 3	CACCTTGGATTGAGAGTCAAGAC	AGGAATCGGCTATATTGCTGGT
HIF1-α	GATGACGGCGACATGGTTTAC	CTCACTGGGCCATTTCTGTGT
c-Myc	CCCTATTTCATCTGCGACGAG	GAGAAGGACGTAGCGACCG
LDHA	CAAAGACTACTGTGTAACTGCGA	TGGACTGTACTTGACAATGTTGG
GLUT1	TCAAACATGGAACCACCGCTA	AAGAGGCCGACAGAGAAGGAA
IL-6	CTGCAAGAGACTTCCATCCAG	AGTGGTATAGACAGGTCTGTTGG
IL-10	CTTACTGACTGGCATGAGGATCA	GCAGCTCTAGGAGCATGTGG
GAPDH	AAGAAGGTGGTGAAGCAGG	GAAGGTGGAAGAGTGGGAGT

### Preparation of SDPN

DPN were prepared by a modified nanoprecipitation method, as described previously [23]. Briefly, LU and SL (molar ratio 1:1) were dissolved in a PLGA acetone solution (6 mg/mL) as the oil phase. The lipids DPCP and DSPE-PEG-2000 were separately dissolved in anhydrous ethanol (100 mg/mL) and then added to deionized water as the aqueous phase at 65 °C. The oil phase was dropped into the water phase at 25 °C, with stirring at 300 rpm for 2 h. The organic solvent was removed, followed by 0.8-µm filtration to obtain luteolin and silibinin co-loaded DPN (LU/SL-DPN).

SDPN were prepared by the association of sophorolipid with DPN. A sophorolipid dispersion was prepared by dispersing sophorolipid and SL in deionized water with a homogenizer (IKA® RCT, Konigswinter, Germany) at 8000 rpm for 30 min. Subsequently, the mixture was filtered using a 0.8-µm membrane. The sophorolipid dispersion was then added into the LU/SL-DPN preparation under gentle stirring before rotary evaporation to obtain luteolin and silibinin co-loaded SDPN (LU/SL-SDPN). LPN, using Lipoid S100 instead of DPCP, was used as control.

### Characterization of nanoparticles

The particle size and zeta potential of nanoparticles were detected using a dynamic light scattering meter (Nano ZS 90, Malvern Instruments, Malven, UK). The morphology of nanoparticles was determined as follows. Nanoparticle samples were dropped onto a copper mesh covered with a support film after about a 20:1 dilution with deionized water. Samples were then dried and negatively stained with 2% phosphotungstic acid solution. The nanoparticle images were captured using transmission electron microscopy (TEM; Tecnai G2 Spirit, USA). The conditions for X-ray diffraction analysis were as follows: radiation source Cu/Kα; current 100 mA; working voltage 40 kV; scanning speed 5°/min. Drug loading and encapsulation efficiency of nanoparticles were measured by ultrafiltration centrifugation using an improved HPLC method. Fourier-transformed infrared (FTIR) analysis was carried out as follows: LU/SL-SDPN, SDPN, DPN, LU, SL, sophorolipid, DPCP, DSPE-PEG2000, and PLGA were measured via FTIR spectroscopy. For each spectrum, the sample was mixed with KBr and tableted to form pellets and then analyzed using a FTIR spectrophotometer (Presitage 21, Shimadzu, Kyoto, Japan) in the spectral range of 400–4000 cm^− 1^.

### In vitro physical stability in simulated gastrointestinal fluid

The stability of nanoparticles in vitro was determined in simulated gastric fluid (SGF) (pH 1.2), simulated intestinal fluid (SIF) (pH 6.8), and PBS (pH 7.4) at 37 ℃. After mixing, the size of the nanoparticles dispersed in SGF, SIF, or PBS (1:9) was measured.

### In vitro cytocompatibility, hemolysis assay and histological safety evaluation

The bio-safety assay was performed by evaluating cytocompatibility, blood compatibility and histological safety of nanoparticle [24]. The methodology of bio-safety assay is provided in the Supporting Material.

### Multiple-particle tracking

A multiple-particle tracking assay was used for investigating the motion track of particles in mucus to discern nanoparticle mucosal-penetrating ability. CdSe/ZnS quantum dot (QD) loaded nanoparticles were prepared, as described above, replacing the drug with the fluorescence probe QD. The trajectories of the nanoparticles in mucus were then detected, as shown in a previous report [7].

### Cellular uptake efficacy

To evaluate the cell membrane interaction advantages of DPCP, coumarin-6-labeled LPN and DPN were used to investigate the cellular uptake efficacy. Caco-2 cells were cultured overnight after seeding in 12-well culture plates (2.5 × 10^5^ cells/well). Firstly, the cells were subjected to equilibration with HBSS for 30 min, followed by addition of coumarin-6-loaded LPN and DPN mixed with the medium (1:9, v/v), followed by incubation for 2 h. The cells were washed three times with PBS, digested with trypsin, centrifuged, and the collected cell suspensions were washed twice with PBS. After adding adequate fresh PBS for dispersion, the samples’ fluorescence intensity was detected using CytoFLEX S flow cytometry (Beckman Coulter, Brea CA, USA) at 488 nm.

The fluidity of the nanoparticle surface was determined using the fluorescent dye laurdan [25]. The generalized polarization (GP) value of laurdan embedded in the nanoparticles was calculated from the fluorescence intensity by a microplate reader using the following equation:

$$GP=\frac{{I}_{440}-{I}_{490}}{{I}_{440}+{I}_{490}}$$,

where I_440_ and I_490_ indicate the emission wavelengths with excitation at 340 nm. The molar ratio of lipid and laurdan in the nanoparticle was approximately 100:1. Then, the nanoparticle dispersion was diluted in PBS (700:1, v/v), followed by incubation at 25℃ for 1 h. LPN and DPN rigidity were assessed by Young’s modulus using an Atomic Force Microscope Dimension Fast Scan Bio (Bruker AXS, Karlsruhe, Germany).

### In vivo intestinal absorption and in situ Peyer’s patch absorption

Sprague–Dawley (SD) rats (female, 200 ± 20 g, clean grade) were used to investigate intestinal and Peyer’s patch absorption. P2 fluorescent probe-labeled DPN and SDPN were used to evaluate nanoparticle absorption in the intestinal tract. After fasting overnight, rats were gavaged with 2 mL of P2-labeled nanoparticles. An hour after administration, rats were anesthetized (10% chloral hydrate, ip), and the intestines were exposed by opening the abdominal cavity along the abdominal midline, followed by the removal of excess dispersion in the jejunum section. Intestinal segments of about 0.5 cm were collected and washed with saline. Intestinal segments were placed on a black 96-well plate to measure the fluorescence intensity at Ex.710/Em.760 nm by IVIS spectrum (Lumina XR, PerkinElmer, Waltham, MA, USA).

To further assess nanoparticle Peyer’s patch absorption, P2-labeled DPN and SDPN were administrated to in situ intestine. Rat intestinal segments containing the Peyer’s patch were exposed under anesthesia. One segment end was ligated, while the other was injected with P2-labeled nanoparticles and then ligated. After incubation for 1 h, the ligated intestinal segments were harvested. Intestinal segments were then cut longitudinally to remove the mucus layer for Peyer’s patch collection. Peyer’s patches were trimmed evenly and placed on a black 96-well plate. The fluorescence intensity was measured by IVIS spectrum (Ex.710 nm/Em.760 nm). The protein in Peyer’s patches were quantified using a BCA kit (Thermo Fisher Scientific, Waltham, MA, USA) according to the instructions.

### Transfer into the mesenteric vascular system

Transfer of nanoparticles into the mesenteric vascular system was studied by a two-photon microscopy system (Nikon A1-SHRM-C, Japan). The SD rats were briefly fasted overnight. After anesthetization with chloraldurate, an incision was made in the abdomen, and the intestines were exposed. The jejunum, approximately 3-cm in length, was ligated at one end and pasted to a glass slide. BODIPY FL Succinimidyl Ester- and P4- loaded nanoparticles were administrated to the incised jejunum, which was then ligated at the other end. The mesentery adjoining the jejunum was then attached to the slide and slightly covered. Thereafter, time-lapse images were obtained at each time point by two-photon microscopy with a water-immersed 16× objective.

### Regulation of Raw264.7 macrophage polarization in vitro

RAW264.7 cells were stimulated with 300 ng/mL LPS for 24 h. The RAW264.7 cells were then treated with LU/SL-DPN or LU/SL-SDPN (1:1 molar ratio of LU to SL, 6 µM). Subsequently, cells were washed, scraped off, and transferred to centrifuge tubes, followed by staining with BV421-MHC-II and PE-CD86 for 30 min. The cells were washed twice and resuspended in flow tubes for flow cytometry.

RAW264.7 cells were stimulated with 60 ng/mL IL-4 in 10% FBS DMEM overnight, followed by DMEM containing 5% FBS for 48 h. Then, the cells were treated with LU/SL-DPN or LU/SL-SDPN (1:1 molar ratio of SL to LU, 6 µM). Subsequently, cells were washed, scraped off, and transferred to centrifuge tubes. Thenceforth, 150 µL 1×Cyto Fix was used for fixing and permeating, followed by incubation for 20 min at 25 ℃, washing with 1×perm washing buffer twice, and staining with APC-anti-mouse CD206 for 30 min. The cells were washed and resuspended to flow tubes for expression measurement by flow cytometry. For CLSM (TCS SP8, Leica, Wetzlar, Germany) observation, the cells were additionally stained with DAPI, placed onto an observation dish and observed at excitation wavelengths of 405, 488, and 638 nm. qPCR assays were conducted to analyze the relative mRNA expression of TNF-α, MR, TGF-β1, and IL-6 in a stimulated M1/M2 Raw264.7 cell model.

### In vitro energy metabolism assays

The 4T1 cells seeded into XF96 microplates (5000 cells/well) were cultured in culture media containing CoCl_2_ (100 μM) overnight at 37 °C, 5% CO_2_. After removal of the culture medium, the cells were incubated with LU/SL-SDPNs, LU/SL-DPNs for 1 h [incubation media containing CoCl_2_ (100 μM)] and then washed with phenol red-free XF DMEM medium. The plate was equilibrated in a 37 °C CO_2_-free incubator for 1 h. Then, a Seahorse XFe96 Analyzer (Agilent, Santa Clara, CA, USA) was used for calibration. The cells were subjected to detect oxygen consumption rate (OCR), glycolytic proton efflux rate (glycoPER), and real-time ATP rate according to the instructions of the kits (XF Cell Mito Stress Test kit, XF Glycolytic Rate Assay kit, XF Real-time ATP Assay kit). After the cell metabolism assay, 4T1 cells were stained by Hoechst 33,342 (Yeasen Biotechnology, Shanghai, China), according to the instructions, and counted by BioTek Cytation 5 (Winooski, VT, USA).

### In vivo anti-breast cancer metastasis studies

Balb/c mice (female, 6¬8 weeks old) were used to establish a 4T1 tumor-bearing model. A 100 µL of 4T1 cell suspension (5.5 × 10^5^/mouse) was subcutaneously injected at the second mammary fat pad of each mouse. They were then randomly divided into five groups (5 mice per group). The mice were treated with either saline, LU-LPN, LU/SL-LPN, or LU/SL-SDPN (80 mg/kg, molar ratio SL:LU = 1:1) by oral gavage every other day. Mice were sacrificed on the 28th day, and the tumor and organs were excised.

Metastatic lung nodules were recorded. The tumor, liver, heart, spleen, lung, kidney, and jejunum were fixed in 4% paraformaldehyde, followed by pathological evaluation. The tumor tissues were also analyzed by immunohistochemistry and immunofluorescence. The tumor tissues were briefly sliced, and the tumor slices were incubated with anti-MMP9, anti-CD31, anti-TGF-β1, anti-IL-6, anti-IL10, anti-CD86, anti-CD206, anti-GLUT1, anti-α-SMA, or anti-collagen I, followed by incubation with secondary antibodies.

Tumor single-cell suspensions were harvested, as previously described [7]. Subsequently, fresh tumor pieces were lysed in an RPMI 1640 medium with collagenase I and DNAase. The homogenates were then filtered to acquire single-cell suspensions dissociated from RBCs. 5–10 × 10^6^ cells/mL were used for further antibody staining. The cells were collected for flow cytometry analysis.

The relative mRNA expression of CD86, CD206, STAT3, HIF1-α, IL-6, IL-10, c-Myc, LDHA, and GLUT1 in tumor tissue was detected by qPCR assays.

### Statistical analysis

Data are shown as mean ± standard deviation (SD). Results analyses were performed by Student’s unpaired *t*-tests or one-way ANOVA. Graphpad Prism software (version 8.0.1, Graph Pad, San Diego, CA, USA) was used for all analyses. *P* < 0.05 was considered statistically significant.

## Results and discussion

### Optimum ratio of LU and SL in combination

**Fig. 1:**
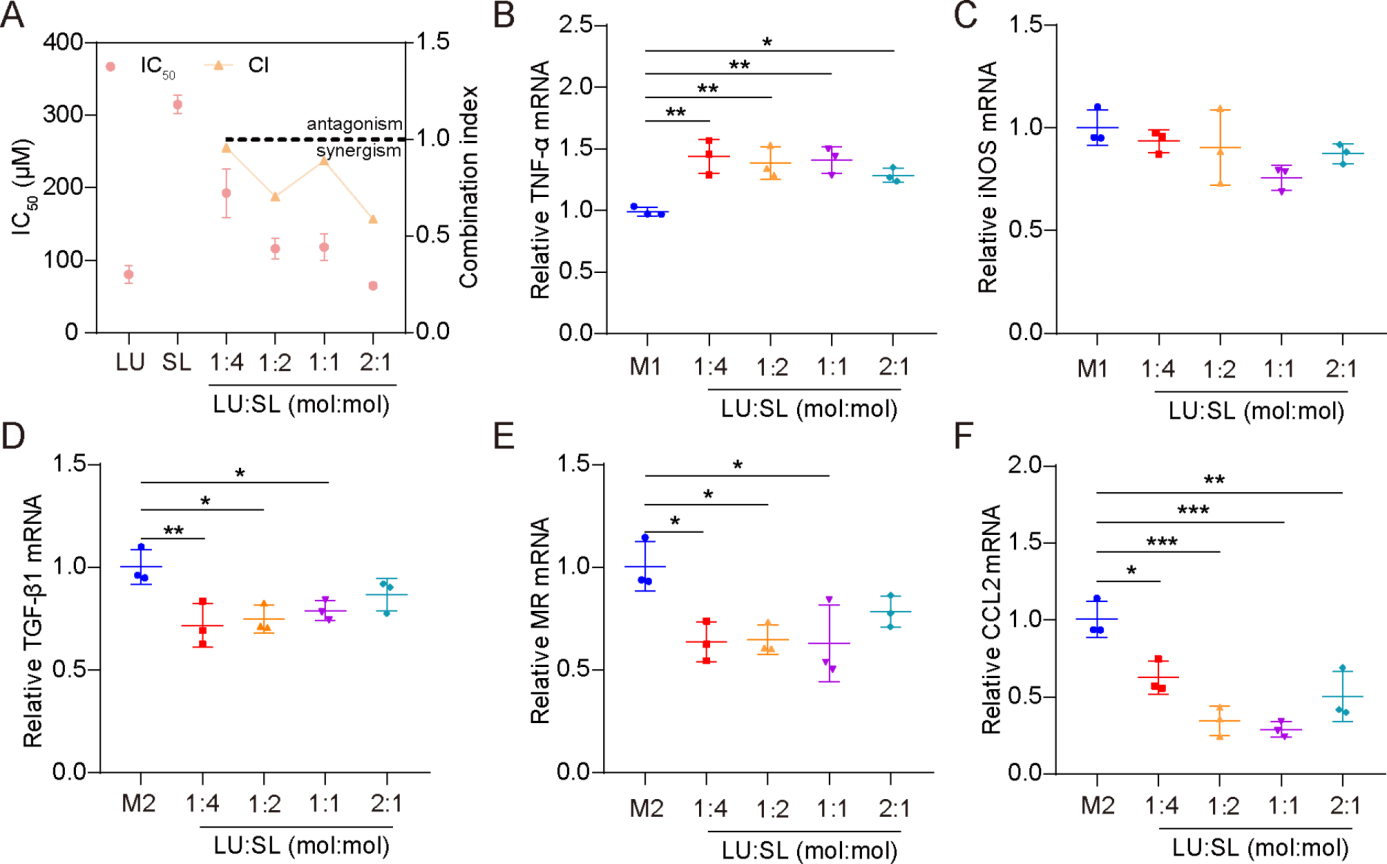
Optimum ratio of a combination of LU and SL, as determined by cell viability and qPCR assays. **(A)** The cytotoxicity (IC_50_) and combination index (CI) in 4T1 cells with different ratios of free LU/SL. **(B-F)** The relative mRNA expression of the macrophage-associated markers TNF-α, iNOS, TGF-β1, MR, and CCL2 by qPCR assay after LU/SL treatment in Raw264.7 cells. Mean ± SD, n = 3; *P < 0.05, **P < 0.01, ***P < 0.005. Abbreviations: LU/SL, luteolin and silibinin; SD, standard deviation; qPCR, quantitative polymerase chain reaction

The synergistic action of LU and SL were evaluated based on cell viability of 4T1 cells and mRNA expression of M1/M2 repolarized Raw264.7 cells. All molar ratio combinations (4:1, 2:1, 1:1 and 1:2) of LU and SL showed a combination index (CI) < 1 (Fig. 1A), demonstrating synergistic effects with increased tumor cytotoxicity [27]. Furthermore, the combination of LU and SL upregulated M1-related mRNA TNF-α (Fig. 1B) or maintained the iNOS level (Fig. 1C) and exhibited an optimized effect on the re-education of M2 macrophages to M1 macrophages, reflected by downregulated M2-related mRNA TGF-β1, MR, and CCL2 (Fig. 1D-F). LU and SL in a 1:1 molar ratio showed favorable synergistic inhibitory effects with a CI of 0.889 as well as optimized macrophage repolarization effects. Therefore, the molar ratio of 1:1 was used as the optimum ratio to fabricate SDPN in this study.

### Characterization of SDPN

**Fig. 2:**
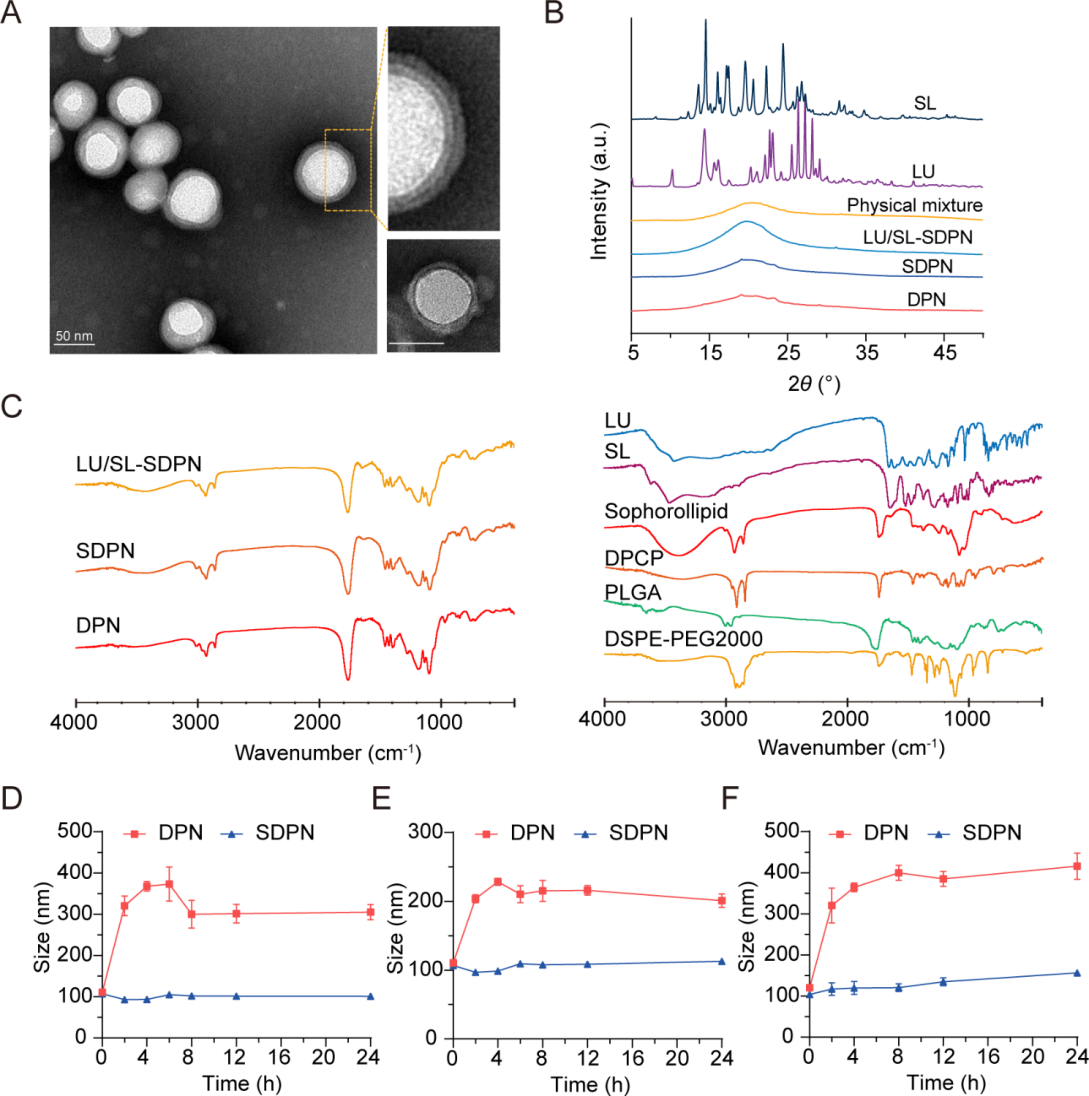
Nanoparticle characterization and nanoparticle physical stability in simulated gastrointestinal fluids. **(A)** Transmission electron microscope image of SDPN (left) and DPNs (lower right corner); Scale bar = 50 nm. **(B)** X-ray diffraction patterns of SL, LU, physical mixture, drug-free DPN, SDPN, and LU and SL co-loaded SDPN. **(C)** FTIR spectra of LU/SL-SDPN, SDPN, DPN (left) and luteolin, silibinin, sophorolipid, DPCP, DSPE-PEG2000, and PLGA (right). **(D, E)** Mean particle size of nanoparticles in simulated gastric fluid **(D)**, simulated intestinal fluid **(E)**, and PBS (pH7.4) **(F)**. Mean ± SD, n = 3. Abbreviations: SDPN, sophorolipid-associated membrane-biomimetic choline phosphate-poly(lactic-co-glycolic) acid hybrid nanoparticle; LU, luteolin; SL, silibinin; FTIR, Fourier-transformed infrared; PLGA, poly(lactic-*co*-glycolic acid)

We fabricated cell membrane-biomimetic lipid polymeric hybrid nanoparticles SDPN by a modified nanoprecipitation method using DPCP and DSPE-MPEG2000 attached to sophorolipids and then evaluated the nanoparticles. The obtained SDPN were less than 100 nm in size (Table 2). They showed a lower absolute zeta potential than the nanoparticles without sophorolipid attachment (DPN). The lipid was coated onto the PLGA core to form the shell-core structure. As the lipid shell is composed of DPCP, the thickness of DPCP on the surface of PLGA is relevant. The encapsulation efficiency of LU and SL in SDPN were 97.1% and 96.5%, respectively, while their drug loadings were 3.49% and 2.04%, respectively. Due to their hydrophobic interactions, LU and SL might mostly be loaded into PLGA core, and several of them were distributed at the interface of lipid DPCP and PLGA of SDPN. The drug loading of SDPN is mainly related to the amount of PLGA; the more PLGA, the more hydrophobic drugs are loaded. Furthermore, the lipid shell is coated on the core surface of PLGA to further protect the drug from leaking out. SDPN displayed a spherical shape with an obvious core-shell structure and exhibited both a uniform particle size distribution and a uniquely-arranged outer layer (Fig. 2A). This was consistent with previous characterizations of association between nanoparticle surfaces and sophorolipids [7]. The results of XRD are shown in Fig. 2B. The XRD spectrum of SL exhibited strong crystal diffraction peaks at 13.06°, 16.08°, 17.44°, 19.6°, 20.26°, 22.28°, and 24.42°, while LU showed peaks at 10.26°, 13.5°, 22.12°, 23.1°, 25.54°, 26.36°, 27.24°, and 28.14°, indicating the crystal form of the two drugs. However, these crystal diffraction peaks disappeared in the patterns of blank DPN, SDPN, and LU/SL-SDPN, indicating the changed crystal state of SL and LU in nanoparticles, which may be molecular or amorphous forms in SDPN. Further, the possible physicochemical interactions between the components in nanoparticles as well as the therapeutic agent loading were evaluated by FTIR analysis. As shown in Fig. 2C, the sharp and large peak at 1760 cm^− 1^ in the FTIR spectra of DPN and SDPN could be ascribed as the C = O stretching vibration overlapping of the peaks of PLGA, DPCP, and DSPE-PEG2000. The peaks at 1455 cm^− 1^ and 1135 cm^− 1^ related to methyl group C-H stretching and C-O-C stretching, respectively, might belong to PLGA. The peak at 2920 cm^− 1^ observed in the DPN spectrum might be described as the methylene group C-H of DPCP. Compared with the DPN spectrum, the stronger peak at 3420 cm^− 1^ of the SDPN spectrum might belong to the specific O-H vibration of sophorolipids. This is evidence that sophorolipids are attached on the surface of SDPN. Encapsulation of luteolin and silibinin were confirmed due to their specific peaks at 1520 cm^− 1^ (aromatic ring), 1610 cm^− 1^ (C = O from central heterocyclic ring), and 3430 cm^− 1^(OH) weakening or disappearing in the LU/SL-SDPN spectrum. The gastrointestinal tract is a complex physiological environment, and factors such as pH, digestive enzymes, ions, and endogenous substances will affect the stability of nanoparticles [1]. Therefore, ensuring the high stability of nanoparticles in the gastrointestinal tract is the primary precondition for oral absorption. There was no significant particle size change for SDPN, indicating that SDPN remained stable in SGF (Fig. 2D) and SIF (Fig. 2E). Furthermore, SDPN also showed stability in PBS at 37℃ (Fig. 2F). Consistent with our previous study [7], the attachment of sophorolipids formed a protected layer due to their unique feature of resisting pH and ion strength variation.

**Table 2:** Particle size and zeta potential of DPN and SDPN.( mean ± SD, n = 3)

Nanoparticles	Mean particle size (nm)	Polydispersity index	Zeta potential (mV)
DPN	96.08 ± 5.04	0.18 ± 0.02	-23.7 ± 1.3
SDPN	86.45 ± 0.66	0.11 ± 0.01	-11.1 ± 0.5

Abbreviations: SD, standard deviation; SDPN, sophorolipid-associated membrane-biomimetic choline phosphate-poly(lactic-co-glycolic) acid hybrid nanoparticle

### In vitro cytocompatibility and hemolysis assay

The cytotoxicity was evaluated to study the in vitro cytocompatibility of DPN and SDPN. The cell viability of DPN at each concentration was above 95% (Fig. S1A). For SDPN, the cell viability was 95.1%, 94.6%, and 90.3% at the concentrations of 0.1, 0.5, 1.0 mg/mL, respectively (Fig. S1B). These results demonstrated the favorable cytocompatibility of DPN and SDPN in vitro.

The hemolysis percentage (HP) of DPN and SDPN were determined to evaluate nanocarrier blood compatibility. The HP of DPN was lower than 3.5%, which showed a significant decrease compared to the positive control group (H_2_O) (Fig. S2A). The HP of SDPN was lower than 3%, also showing a significant decrease with the positive control group (Fig. S2B). The HP was in accordance with the permissible level of 5%, which indicated their negligible hemolysis and favorable safety for in vivo study.

### Mucus diffusion and in vitro cellular uptake

**Fig. 3:**
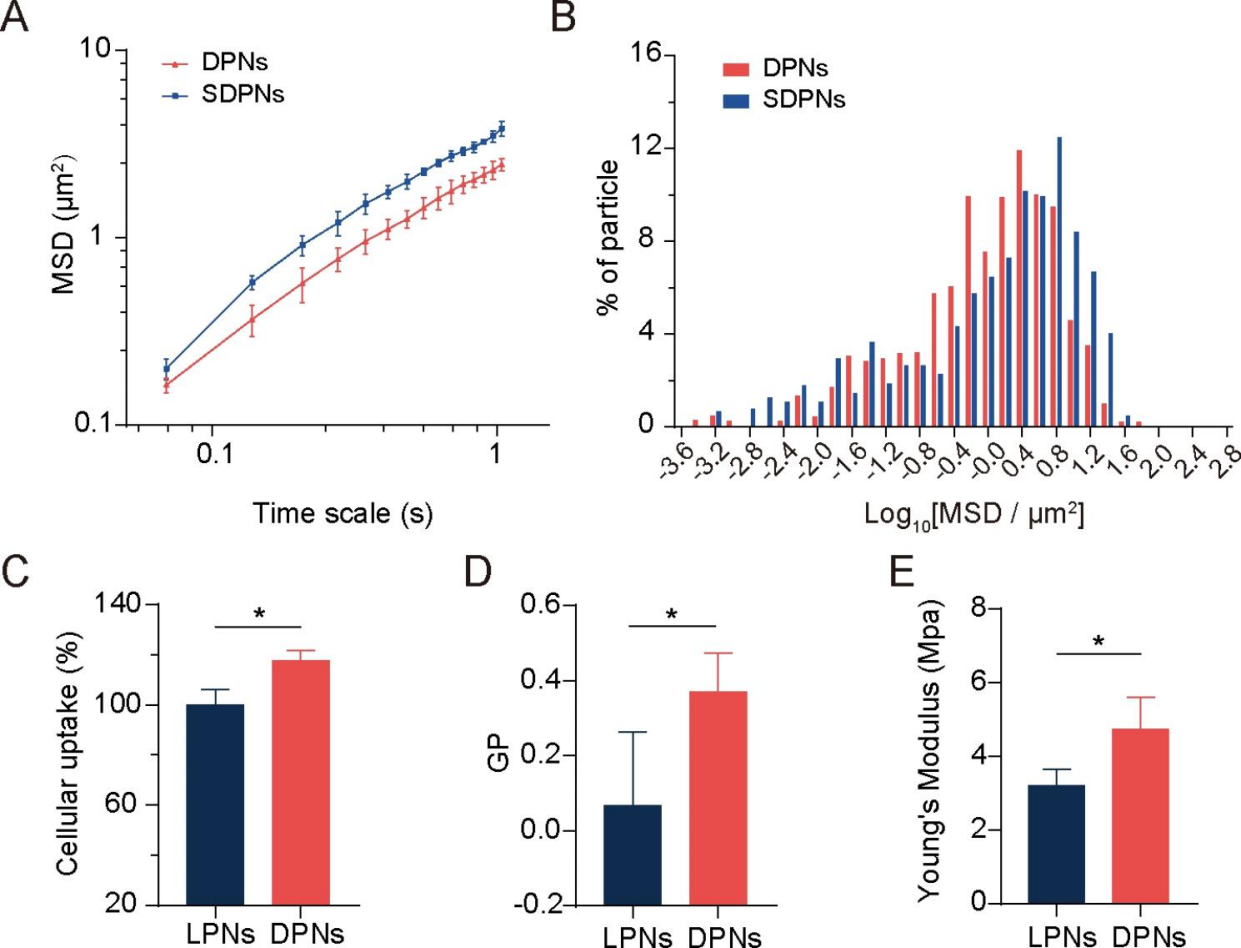
Mucus diffusion and cellular uptake studies. **(A)** Mean square displacement (MSD)–time interval curve for the nanoparticles. **(B)** Histogram of MSD distribution of nanoparticles on a 1.035-s time scale. **(C)** Cellular uptake of LPN and DPN in Caco-2 cells. **(D)** Generalized polarization (GP) value of LPN and DPN. **(E)** The Young’s modulus of LPN and DPN. Mean ± SD, n = 3; *P < 0.05. Abbreviations: SD, standard deviation; LPN, lipid-polymer nanoparticles; DPN: choline phosphate-poly(lactic-co-glycolic) acid hybrid nanoparticle

We evaluated the mucus diffusion of nanoparticles through multiple-particle tracking assays. The larger mean square displacement (MSD) value (Fig. 3A) and higher percentage of particles with high mobility (Fig. 3B) demonstrate the good mucus permeation ability of SDPN. SDPN has hydrophilic and near-neutral surface properties due to the attachment of sophorolipids that improve their mucus penetration capacity. Furthermore, sophorolipid assemblies may dissociate from the surface of nanoparticles during mucus transit due to their weak interaction with the outer lipid fraction of nanoparticles, according to our previous study [7], which allows the exposure of DPCP to generate endocytosis by enterocytes.

The membrane-biomimetic strategy was expected to improve the cellular uptake capacity of DPN. To investigate this hypothesis and evaluate the benefits of the cell membrane interaction of DPCP to nanoparticles uptake, we used nanoparticle LPN with PC cover instead of DPCP as a control. Compared with LPN, DPN significantly improved the cellular uptake rate in Caco-2 cells (Fig. 3C), indicating a higher internalization efficiency. Cell membrane phospholipids contain a zwitterionic PC head. DPCP, as reported in a previous study [2], was synthesized with three parts: headgroups (hydrophilic head composed of PC-reversed choline and phosphate groups), a C16 alkane tail, and a linker. The synthesized hydrophilic head in DPCP conferred a charge direction reversal and was considered to provide biorthogonality in the backbone. In a previous study [24], CP exhibited cell adhesion action and protein resistance due to its natural zwitterionic properties and supramolecular ionic pair interactions with cell membranes. In the current study, as illustrated in Scheme 1 A, when DPN (available post SDPN mucus diffusion) contacted an enterocyte, the CP-PC interaction may have occurred via supramolecular ion pair (-(CH_3_)_3_ N^+^—PO_3_^−^) with PO_3_^−^—(CH_3_)_3_ N^+^). The interaction with cell membranes through CP-PC promoted the adhesion of nanoparticles to enterocytes and improved the affinity of nanoparticles with cell membranes, leading to efficient nanoparticle cell internalization. The biomimetic application of DPCP in SDPN delivery provided a universal binding mechanism to attach nanoparticles to enterocyte membranes. Several factors also influence the uptake behavior and efficiency of nanocarriers [28], such as size, surface properties, fluidity, rigidity, and ligand properties of surface modifications. As DPN and LPN have similar surface charges and particle sizes, it suggested that the size and charge of nanoparticles might not be the main causes of their differences in cellular uptake. Thus, we measured the fluidity of nanoparticle membrane containing lipids. The GP values of DPN were significantly higher than those of LPN (Fig. 3D), demonstrating that DPN were less fluid compared with LPN. The surface fluidity of lipids has been proven to facilitate interaction with cells due to increased opportunities for nanoparticles to contact and fuse with cell membranes [29]. When nanoparticles are endocytosed, they encounter two opposing forces. One is the attractive force driving nanoparticles and cells together, comprising van der Waals forces, electrostatic action, and ligand-receptor interactions. The other is a repelling force that prevents nanoparticles from entering the cell, affecting wrapping of nanoparticles, bending of the cell membrane, and membrane tension. However, the greater fluidity of the nanoparticle lipid layer means more energy is required to overcome the bending change in the cell membrane as well as the encapsulation of the nanoparticles, facilitating internalization of more fluid nanoparticles. The larger the wetting angle of the nanoparticles, the greater the extent of nanoparticles unfolding on the surface of the cell membrane needed [30]. The bending of energy barriers that must be overcome is related to the extent of the nanoparticles’ spread. Suitable membrane fluidity can balance these two opposing forces, which is conducive to maximizing internalization efficiency. Therefore, DPN possessed more favorable lipid layer fluidity than LPN. We also measured the Young’s modulus of nanoparticles by atomic force microscopy to estimate the particles’ overall rigidity. The Young’s modulus of DPN was significantly higher than that of LPN (Fig. 3E), indicating that DPN might stay rigid. Deformation is not apparent when entering the cell membrane, partly explaining its accessibility for cellular internalization [25]. It has been shown that nanoparticles with moderate rigidity not only exhibit enhanced diffusion in mucus, but also overcome the intestinal barrier [31]. Soft nanoparticles exhibit weak mucus penetration and low cellular uptake due to their excessive change and irregular shape. Hence, spherical nanoparticles with a certain stiffness tend to deform into ellipsoids in a complex mucus network structure, promoting rapid penetration and exhibiting better cellular uptake capability. The suitable fluidity and overall rigidity of nanoparticles might be well provided by the DPCP as the lipid component of DPN, affecting the nanoparticle surface property and being beneficial to cellular uptake.

### In vivo intestinal absorption and transport

**Fig. 4:**
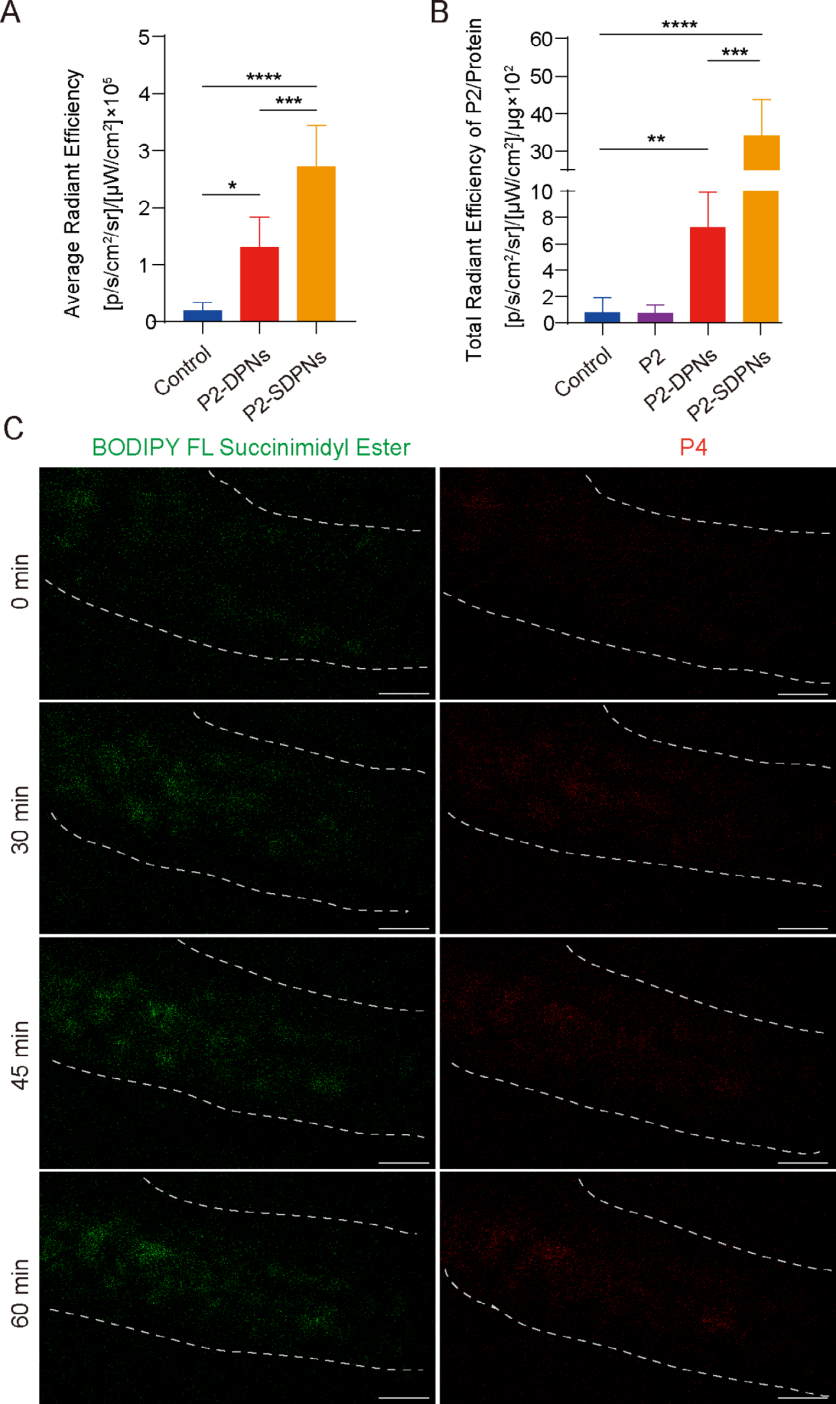
Nanoparticle intestinal and Peyer’s patch absorption and mesenteric vascular transport in situ in the rat. **(A)** Fluorescence intensity analysis of DPN and SDPN in jejunum after oral administration for 1 h. **(B)** Fluorescence intensity of DPN and SDPN per unit of Peyer’s patch protein after oral administration for 1 h. Mean ± SD, n = 3; *P < 0.05, **P < 0.01, ***P < 0.001, ****P < 0.0001. **(C)** Transportation of SDPN into mesenteric vascular tissue after administration for 0, 30, 45, and 60 min imaged by two-photon microscopy. The mesenteric blood vessel area was circled by the dotted line. Scale bar = 100 μm. Abbreviations: SDPN, sophorolipid-associated membrane-biomimetic choline phosphate-poly(lactic-co-glycolic) acid hybrid nanoparticle; SD, standard deviation

The fate of SDPN in the gastrointestinal tract was further revealed by detecting the in vivo intestinal absorption and in situ Peyer’s patch absorption as well as the transfer into the mesenteric vascular system. To track intact nanoparticles labeled with fluorescent probes, a P2 probe was used for nanoparticle oral intestinal absorption studies. P2 is a class of near-infrared fluorescence aza-BODIPY probes, emitting stable and strong fluorescence when carried in hydrophobic cores of nanoparticles [32, 33]. Compared with DPN, the fluorescence intensity of SDPN was significantly increased (Fig. 4A), indicating that SDPN improved absorption by the intestinal epithelium, consistent with the quick permeation through the mucus layer and improved cell uptake in vitro. Peyer’s patch absorption of SDPN in rats was also significantly increased (Fig. 4B), which was expected for the lymphatic pathway absorption of nanoparticles. Furthermore, the transport of SDPN into mesenteric vessels was observed by two-photon microscopy in situ in rats. SDPN are loaded with the fluorescent probe BODIPY FL Succinimidyl Ester (green signal) in the associated sophorolipid and P4 (red signal) in the core. SDPN showed distinct fluorescence in mesenteric vessels at 30, 45, and 60 min (Fig. 4C) compared to 0 min, suggesting that a portion of the nanoparticles were transported intact through the mesenteric vessels and absorbed into the blood.

### In vitro regulation of macrophage polarization

**Fig. 5:**
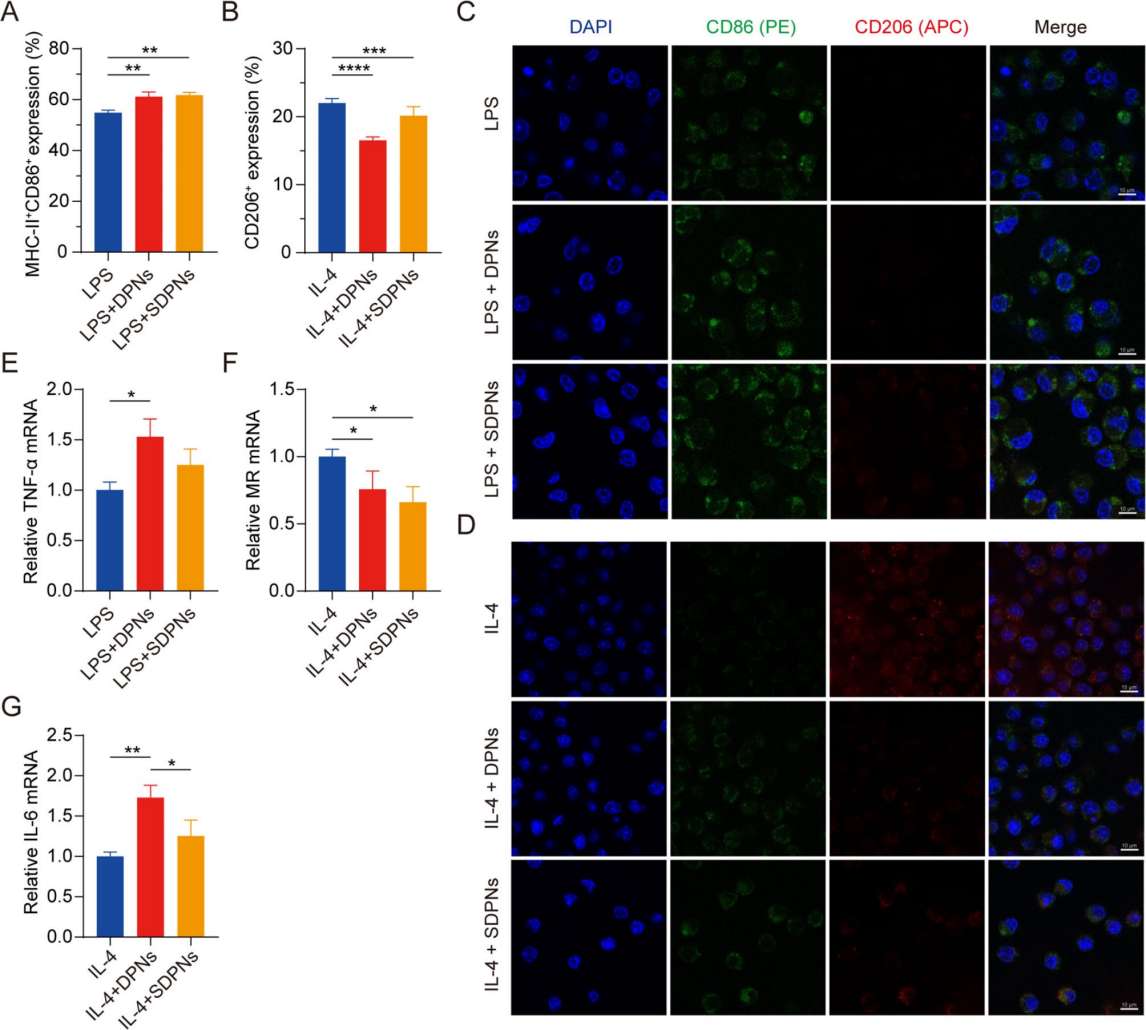
In vitro regulation of macrophage repolarization by nanoparticles in Raw264.7 cells. (A, B) Flow cytometry measurement of MHC-II^+^CD86^+^ or CD206^+^ expression after treatment with nanoparticles in M1 macrophages (300 ng/mL LPS induction) or nanoparticles in M2 macrophages (60 ng/mL IL-4 induction). (C, D) Laser confocal image of nanoparticles on M1/M2 macrophage. Blue fluorescence: cell nuclei by DAPI; Green fluorescence: CD86; Red fluorescence: CD206. Scale bar = 10 μm. (E-G) The relative mRNA expression of the macrophage-associated markers TNF-α, MR, and IL-6 by qPCR assay. Mean ± SD, n = 3; *P < 0.05, **P < 0.01, ***P < 0.005, ****P < 0.001. Abbreviations: qPCR, quantitative polymerase chain reaction; SD, standard deviation; DAPI, 4′,6-diamidino-2-phenylindole; LPS, lipopolysaccharide

To investigate the role of co-loaded nanoparticles in regulating M1 and M2 macrophage polarization, we evaluated the expression of the macrophage protein biomarkers CD86, MHC-II, and CD206 by flow cytometry with M1 or M2 RAW264.7 cells, induced by LPS or IL-4, respectively. The expression of CD86^+^ MHC-II^+^ in the LPS + DPN and LPS + SDPN groups was significantly increased compared with the LPS group (Fig. 5A), indicating that the nanoparticles could promote M1 phenotype macrophages. The CD206^+^ expression in the IL-4 + DPN group decreased significantly compared with the IL-4 induction group (Fig. 5B), indicating that nanoparticles could suppress M2 macrophages. In addition, compared with the IL-4 + SDPN group, the expression of CD206^+^ in the IL-4 + DPN group was lower, which might be due to the different surface properties of nanoparticles giving rise to different macrophage regulation mechanisms or cellular uptake abilities [34].

As shown in the CLSM images (Fig. 5C and D), SDPN increased green fluorescence signal intensity (CD86) in the in vitro M1 macrophage model induced by LPS, whereas the red signal intensity (CD206) remained unchanged. In M2 macrophages, SDPN remarkably decreased the red fluorescence intensity while generally increasing the green one. Consistent with the flow cytometry results, the CLSM observations demonstrated that SDPN upregulated M1-related CD86 levels and downregulated M2-related CD206 levels.

In qPCR assays, Fig. 5E and F show that the repolarization effect of the nanoparticles is demonstrated by the notably increased levels of the M1-related anti-tumor cytokine TNF-α, while decreased relative mRNA levels of the M2-related biomarker MR. In addition, the expression of IL-6 was upregulated (Fig. 5G); this upregulation is related to M1 macrophages, indicating the repolarization of M2 to the M1 macrophage phenotype. Collectively, these findings suggested that DPN and SDPN regulated the Raw264.7 macrophage phenotype, promoting M1 phenotype macrophages and reducing M2 phenotype macrophages, and implying that there was a polarization effect of nanoparticles on TAM from the tumor-promoting to the anti-tumor phenotype.

### In vitro regulation of OXPHOS and glycolysis

**Fig. 6:**
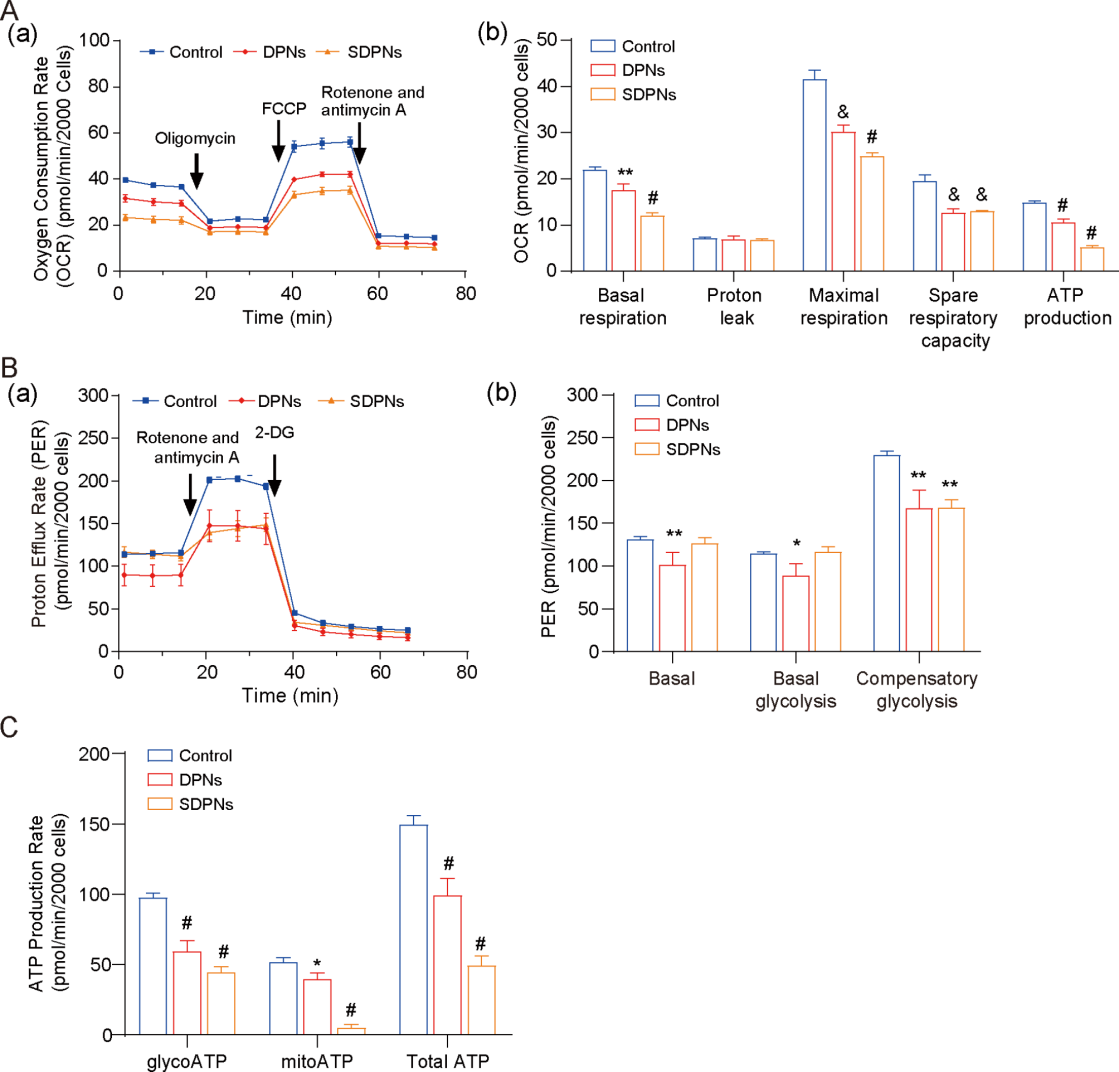
In vitro nanoparticle regulation of metabolism in 4T1 cells under hypoxic conditions: **(A)** Mito pressure test. **(a)** OCR curves in 4T1 cells treated with oligomycin, FCCP, and both rotenone and antimycin A. **(b)** OCR values of basal respiration, maximum respiration, spare respiratory capacity, and ATP production treated with LU/SL-DPNs and LU/SL-SDPNs for 1 h. **(B)** Glycolytic rate test. **(a)** PER curves in 4T1 cells treated with 2-DG and both rotenone and antimycin A. **(b)** PER values of basal proton efflux rate, glycolytic proton efflux rate, and compensatory glycolysis treated with LU/SL-DPNs and LU/SL-SDPNs for 1 h. **(C)** Real-time ATP test for glyco ATP, mito ATP, and total ATP treated with LU/SL-DPNs and LU/SL-SDPNs for 1 h. (mean ± SD, n = 3) Compared with the control group. *P < 0.05, **P < 0.01, &*P* < 0.005, #*P* < 0.001. Abbreviations: OCR, oxygen consumption rate; SDPN, sophorolipid-associated membrane-biomimetic choline phosphate-poly(lactic-co-glycolic) acid hybrid nanoparticle; qPCR, quantitative polymerase chain reaction; SD, standard deviation; LU/SL, luteolin and silibinin

4T1 is a rapidly-proliferating and highly invasive cell line that exhibits high metabolic plasticity (metabolic reprogramming ability), easily switching between OXPHOS and glycolysis to coordinate the production of ATP and the supply of biosynthetic precursors in response to environmental stress [35]. For the investigation of LU/SL-SDPN affecting the metabolic adjustment in breast cancer cells, the hypoxic 4T1 cell model was used. The OCR of 4T1 cells treated with SDPN (Fig. 6A) were significantly decreased versus control, confirming that DPN and SDPN could significantly inhibit oxidative phosphorylation (OXPHOS) in 4T1 cells. In addition, DPN significantly reduced the proton efflux rate (PER), including basal PER, glycoPER, and compensatory glycolysis PER (Fig. 6B). SDPN reduced the compensatory glycolysis PER. These results suggested that SDPN could inhibit glycolysis in 4T1 cells. Furthermore, SDPN significantly inhibited mitoATP and glycoATP production (Fig. 6C). Together, SDPN inhibited the energy metabolism of 4T1 cells by inhibiting both glycolysis and OXPHOS, implying that the regulation of metabolic energy provided support to breast cancer metastasis.

### In vivo antimetastatic effect

**Fig. 7:**
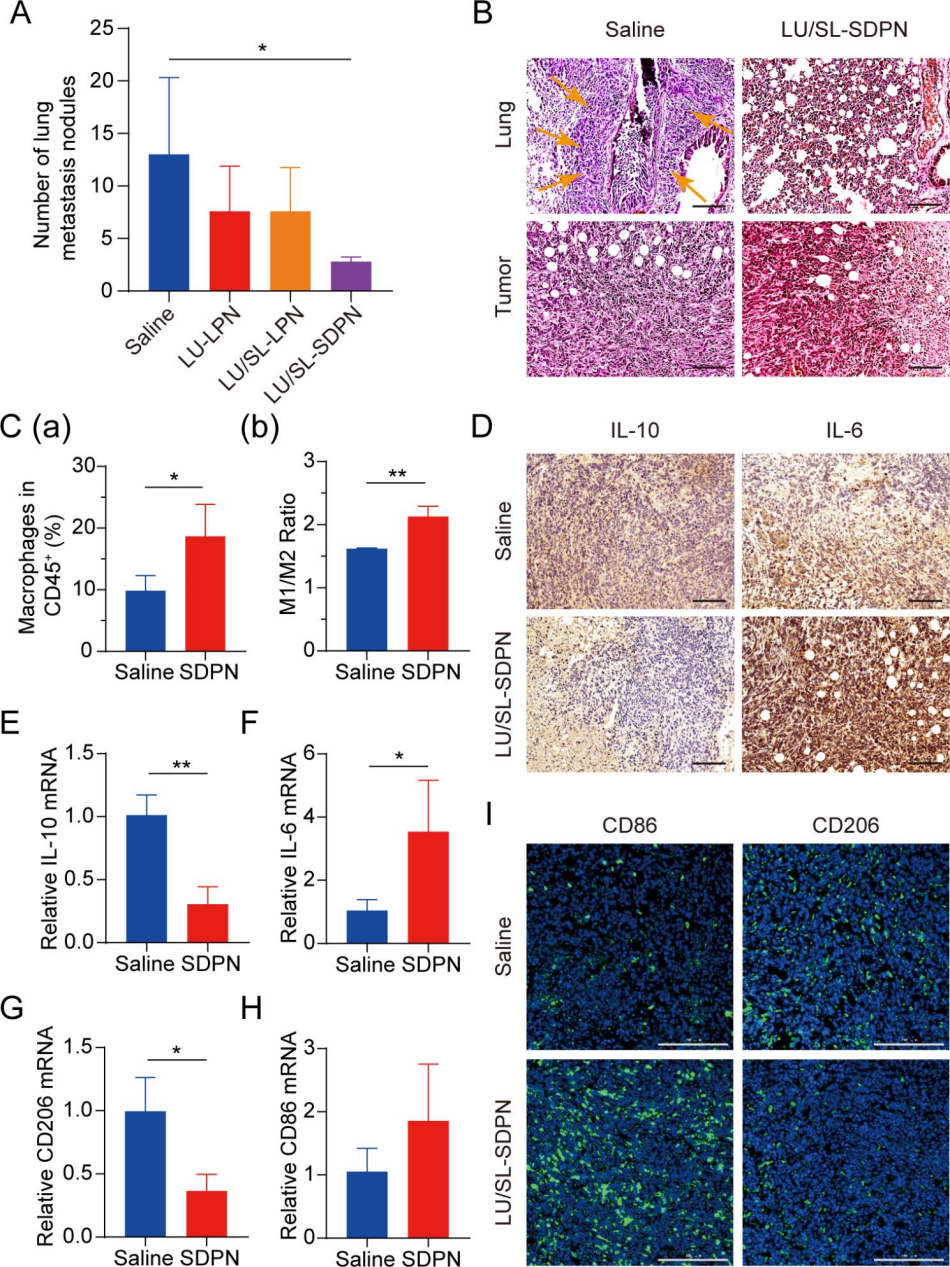
Antimetastatic activity of nanoparticles in vivo. **(A)** Number of lung metastasis nodules from 4T1 tumor-bearing mice in each treatment group. **(B)** H&E staining of tumor and lung tissue sections. Arrows point to the area of intrapulmonary metastasis of tumor cells. Scale bar = 100 μm. **(C)** Analysis of macrophages in 4T1 tumors by flow cytometry. Total macrophages proportion **(a)** and M1/M2 ratios **(b)** of saline and SDPN group-treated tumor tissues. **(D)** Immunohistochemical staining of IL-10 and IL-6 in tumor tissue sections of saline and SDPN groups. Scale bar = 100 μm. **(E-H)** The relative mRNA expression of the macrophage-associated markers IL-10, IL-6, CD206, and CD86 in tumors by qPCR assay. **(I)** Immunofluorescence staining of tumor CD86 and CD206 in tissue sections. Scale bar = 200 μm. Mean ± SD, n = 3; *P < 0.05, **P < 0.01. Abbreviations: SDPN, sophorolipid-associated membrane-biomimetic choline phosphate-poly(lactic-co-glycolic) acid hybrid nanoparticle; qPCR, quantitative polymerase chain reaction; SD, standard deviation; H&E, hematoxylin and eosin

Balb/c mice bearing 4T1 tumor cells were used to assess the effects of LU/SL-SDPN in vivo on the alleviation of breast cancer progression and metastasis. The LU/SL-SDPN-treated group had a significantly reduced number of metastatic pulmonary nodes compared with the saline-treated group (*P* < 0.05) (Fig. 7A). Moreover, H&E staining of lung tissue demonstrated the obvious intrapulmonary metastasis of malignant tumor cells with destroyed alveoli in the saline-treated group, while the LU/SL-SDPN-treated group showed thickened alveoli septa (Fig. 7B). The results of H&E staining of tumor tissues from the saline-treated group showed that tumor cells invaded and infiltrated surrounding tissues, but tumor tissues of the LU/SL-SDPN-treated group were partly necrotic and showed inflammatory cell infiltration. These results indicated that LU/SL-SDPN alleviated breast cancer lung metastasis.

**Fig. 8:**
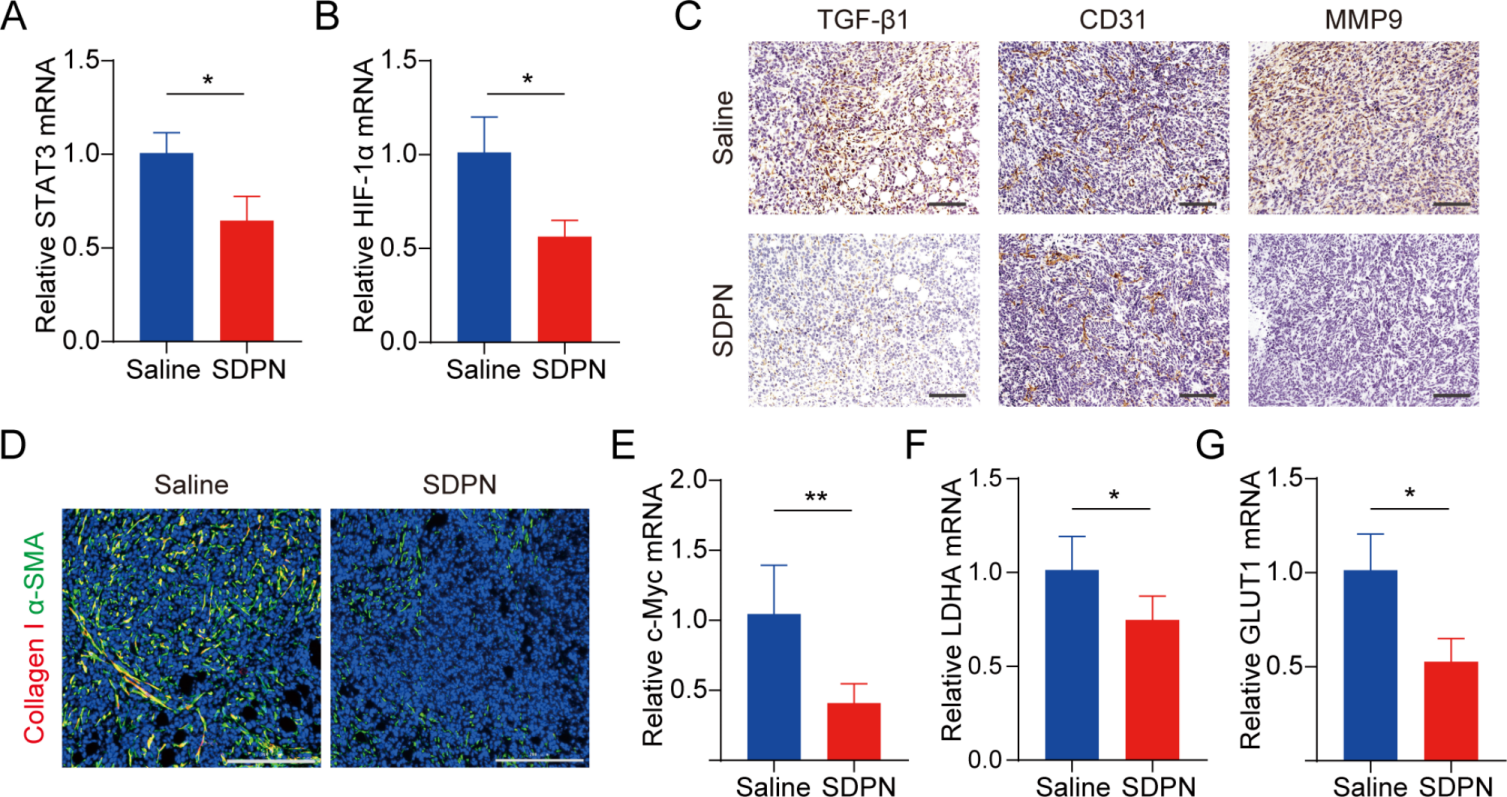
TME and metabolism regulation in 4T1 tumor-bearing mice treated with SDPNs. **(A, B)** The relative mRNA expression of STAT3 and HIF1-α in tumor tissues by qPCR assay. **(C)** Immunohistochemical staining of TGF-β1, CD31, and MMP-9 in tumor tissue sections of saline and SDPN-treated groups. Scale bar = 100 μm. **(D)** Immunofluorescence staining of tumor tissue sections for collagen I and α-SMA. Scale bar = 200 μm. **(E-G)** The relative mRNA expression of c-Myc, LDHA, and GLUT1 in tumor tissues by qPCR assay. Mean ± SD, n = 3; *P < 0.05, **P < 0.01. Abbreviations: TME, tumor microenvironment; SDPN, sophorolipid-associated membrane-biomimetic choline phosphate-poly(lactic-co-glycolic) acid hybrid nanoparticle; qPCR, quantitative polymerase chain reaction; SD, standard deviation

We evaluated LU/SL-SDPN regulation of TAM by analyzing the related protein expression in tumor tissues to reveal the underlying mechanism. Compared with the saline group, macrophage (F480^+^CD11b^+^) proportion in total lymphocytes increased by 8.8% (Fig. 7C(a)). The ratio of the number of MHC-II^+^ cells to CD206^+^ cells in the LU/SL-SDPN-treated group (i.e., the M1/M2 ratio) significantly increased (Fig. 7C(b), S3). Consistently, immunohistochemical staining (Fig. 7D), qPCR (Fig. 7E-H), and immunofluorescence staining (Fig. 7I, S4A) results in the LU/SL-SDPN-treated group showed upregulation of the M1-related cytokine IL-6 and biomarker CD86 and downregulation of M2-related IL-10 and CD206. Together, these results indicated that LU/SL-SDPN promoted the M1 phenotype and inhibited the M2 phenotype, thereby regulating TAM to M1-like macrophage polarization and improving anti-tumor-metastasis effects. The strategy of regulation of TAM to M1 macrophages exploits the impact of M1 macrophages on tumor inhibition, metastasis, and immune activation, by presenting specific protein and secreting cytokines in TME while reducing macrophage elimination. LU has shown the potential to regulate macrophage polarization. LU reduced gene expression in M2 macrophages by inhibiting the phosphorylation of STAT proteins [36]. STAT3 is an important intrinsic pathway that intervenes with TAM polarization [26, 37]. With the elimination of the STAT3 signaling pathway, TAM polarization to the M2 phenotype was reduced, thus inducing anti-tumor immunity [38]. Furthermore, tumor hypoxia affects macrophage polarization. The upregulation of HIF1-α in tumor hypoxia contributes to a higher distribution of M2 macrophages, promoting angiogenesis. Consistently, LU has been reported to inhibit VEGF expression in M2-like TAM and to inhibit activation of HIF1-α and STAT3 phosphorylation signal transduction under hypoxic conditions [19, 36]. Herein, the expression of STAT3 and HIF1-α was significantly downregulated (Fig. 8A-B), suggesting the therapeutic potential of LU/SL-SDPN through the co-action of STAT3 and HIF1-α inhibition, to alleviate breast cancer lung metastasis by reducing the reset of M2 macrophages and increasing M1 macrophages.

It has been reported that TAM participates in regulating the EMT process, promotes angiogenesis, and degrades the extracellular matrix with MMP and other pathways, thus promoting tumor metastasis [39, 40]. Therefore, we further investigated the changes in EMT, angiogenesis, and the ECM treated with SDPN. LU/SL-SDPN was shown to have downregulated the expression of TGF-β1, CD31, and MMP9 by immunohistochemistry staining (Fig. 8C), indicating that LU/SL-SDPN could suppress cancer-associated fibroblasts, alleviate the EMT, and reduce angiogenesis and ECM degradation to regulate the TME. In addition, LU/SL-SDPN downregulated the fluorescence colocalization expression of collagen I and α-SMA in tumor tissue (Fig. 8D, S4B), indicating that the nanoparticles could to some extent reduce fiber formation at the tumor site to inhibit the matrix barrier, helping improve the penetration and accumulation of nanoparticles in tumor tissues and further improving inhibition of breast cancer metastasis. Except for the contribution of TAM regulation, SL affects the regulation of TME and hampers breast cancer invasion and metastasis [8, 41]. SL has also been shown to inhibit angiogenesis [42] and the cytokine TGF-β1[43]. The TGF-β pathway is closely related to the activation of cancer, which enhances profibrotic signaling transduction and changes the ECM composition, constituting a complex matrix environment in tumor tissues [44], wherein excessive collagen fibers interlace to form a reticular barrier, which may isolate immune cells from the tumor tissue, while also affecting drug penetration and delivery [45].

mRNA expression of c-Myc, GLUT1, and LDHA were downregulated in LU/SL-SDPN-treated tumor tissues (Fig. 8E-G), consistent with the fluorescence intensity of the GLUT1 protein, shown in Fig. S4C; this suggests the downregulation of OXPHOS and glycolysis metabolism-related gene expression. Mitochondrial STAT3 promotes OXPHOS through the electron transport chain, while STAT3 in the nucleus can promote aerobic glycolysis, even though the enhancement of glycolysis is not only influenced by STAT3 but also by HIF1-α and the proto-oncogene MYC [46]. c-Myc transcription factor, one of the products of HIF-1α and Myc, regulates aerobic glycolysis of tumor cells by increasing the transcription of common targets, such as GLUT1 and LDHA [47]. The inhibition of the overexpression of STAT3, HIF-1α, c-Myc, GLUT1, and LDHA indicated that SDPN can inhibit breast cancer metastasis by targeting the potential signals mentioned above.

H&E staining results showed no significant organ abnormalities of the liver, heart, spleen, kidney, and jejunum in the saline or LU/SL-SDPN-treated groups, except for the presence of tumor cell metastases in the liver, which was anticipated with the tumor-bearing mice model. Moreover, SDPN did not lead to in vivo organ toxicity, indicating the histological safety of SDPN (Fig.S5).

**Fig. 9:**
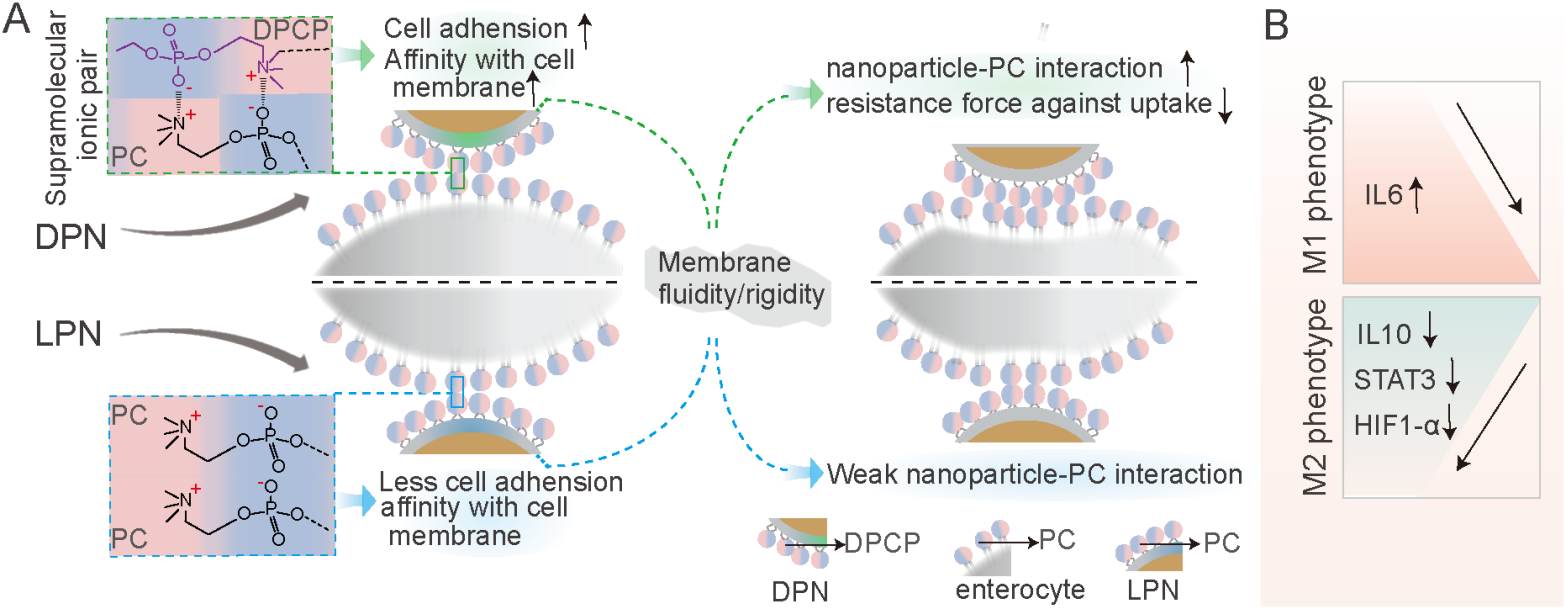
Schematic showing the promoting endocytosis of DPN available at the surface of enterocyte (after SDPN transferred through the mucus layer) **(A)**; regulation of tumor-associated macrophage polarization treated with luteolin- and silibinin-co-loaded SDPN **(B)**. Abbreviations: SDPN, sophorolipid-associated membrane-biomimetic choline phosphate-poly(lactic-co-glycolic) acid hybrid nanoparticle; DPN: choline phosphate-poly(lactic-co-glycolic) acid hybrid nanoparticle

The current study demonstrated that SDPNs confer benefits in enhancing endocytosis and the regulation of TAM (Fig. 9). The endocytosis promotion is summarized as follows: when DPN available at the surface of enterocyte (after SDPN transferred through the mucus layer), the supramolecular interaction provided unique cell adhesion and affinity with the cell membrane. Then, the modified membrane fluidity and rigidity of DPNs favored the DPCP-PC interaction, and possibly reduced the resistance force against endocytosis. The improved oral delivery efficiency of the bioactive ingredients of LU and SL enhanced the action in regulation of TAMs polarization. LU/SL-SDPNs upregulated cytokines IL-6 and downregulated IL-10 in tumor tissues. Additionally, LU/SL-SDPNs inhibited the action of STAT3 and HIF1-α, which were considered to be the important transcription factors involved in M2 polarization.

## Conclusions

In summary, a membrane-biomimetic SDPN was developed for co-loading luteolin and silibinin, STAT3, and HIF 1-α inhibitors to alleviate breast cancer metastasis. SDPN significantly improved cellular uptake in Caco-2 cells by virtue of the supramolecular interaction of DPCP with the cell membrane. In addition, the developed nanoparticles significantly improved oral intestinal absorption and Peyer’s patch absorption. Furthermore, SDPN demonstrated a functional M2-to-M1 macrophage polarization effect and OXPHOS and glycolysis metabolism inhibition in 4T1 cells under hypoxic conditions. In the in vivo 4T1 tumor-bearing mouse model, our results suggested that nanoparticles regulated TAM and TME, which contributed to the alleviation of breast cancer lung metastasis. Therefore, we expect these oral nanoparticles will provide an alternative strategy for aiding TME-related therapy for breast cancer metastasis. However, to further improve oral delivery, efficiency, and other influencing factors, the in vivo mechanisms of cancer metastasis need to be further considered and investigated.

## Electronic supplementary material

Below is the link to the electronic supplementary material.

Supplementary Material 1

## Data Availability

All data are included in this manuscript.
