# Supplementary material for: Oral membrane-biomimetic nanoparticles for enhanced endocytosis and regulation of tumor-associated macrophage
Source: J Nanobiotechnology. 2023 Jul 4;21:206. doi: 10.1186/s12951-023-01949-5 (PMC10318786; doi:10.1186/s12951-023-01949-5)
Supplement: Supplementary file 1 — Supplementary Material 1 [file 12951_2023_1949_MOESM1_ESM.docx]

**Supporting information**

Oral membrane-biomimetic nanoparticles for enhanced endocytosis and regulation of tumor-associated macrophage

Xiaoyan Gu ^a,1^, Rongguang Zhang^a,1^, Yingwei Sun ^a^, Xinyi Ai ^a^, Yu Wang ^a^, Yaqi Lyu ^a^, Xiaoyu Wang ^b^, Yihan Wu ^a^, Zhi Wang ^a^, Nianping Feng ^a,^*, Ying Liu ^a,^*

^a^ *School of Pharmacy, Shanghai University of Traditional Chinese Medicine，Shanghai 201203, C* *b. ^b^Experiment Center for Science and Technology, Shanghai University of Traditional Chinese Medicine, Shanghai, 201203, China*

***Corresponding author**

Prof. Nianping Feng, School of Pharmacy, Shanghai University of Traditional Chinese Medicine, 1200 Cailun Road, Zhangjiang Hi-Tech Park, Pudong New District, Shanghai 201203, P R China. Tel. & Fax: + 86 21 5132 2198; Email: npfeng@shutcm.edu.cn

Dr. Ying Liu, School of Pharmacy, Shanghai University of Traditional Chinese Medicine, 1200 Cailun Road, Zhangjiang Hi-Tech Park, Pudong New District, Shanghai 201203, P R China. Tel.: + 86 21 51322210，Fax: + 86 21 5132197; Email: [debbyly@shutcm.edu.cn](mailto:debbyly@shutcm.edu.cn)

^1^ These authors contributed equally to this work

**Methods**

***In vitro* cytocompatibility study**

The *in vitro* compatibility study was performed to evaluate the compatibility of DPN and SDPN as oral nanomaterials. DPN and SDPN were dispersed in DMEM to form solutions of 0.1, 0.5, and 1.0 mg/mL, respectively. The CCK8 assays were conducted to evaluate the cell viability of nanoparticles treated Caco-2 cells.

**Hemolysis assay**

The blood of healthy SD rats was obtained to separate red blood cells (RBCs) via centrifugation at 3000×*g* for 5 min. RBCs were then washed with PBS (pH 7.4) and diluted with PBS. Thereafter, 0.1 mL of RBCs was added into 0.3 mL of DPN or SDPN (1, 0.5, and 0.1 mg/mL). The samples were incubated at 37℃ for 2 h, and the supernatant was collected via centrifugation at 4000×*g* for 5 min. The absorbance was determined by a microplate reader at 541 nm. The hemolysis percentage (HP) was calculated using the following equation:

$HP(\%)=\frac{{OD}_{t}-{OD}_{neg}}{{OD}_{pos}-{OD}_{neg}}\times100$,

where OD_t_, OD_neg_, and OD_pos_ means the absorption of sample, negative control (RBCs treated with PBS), and positive control (RBCs treated with deionized water), respectively.

**H&E assay**

The liver, heart, spleen, kidney, and jejunum were treated with saline, and LU/SL-SDPN were collected and stained with hematoxylin and eosin.


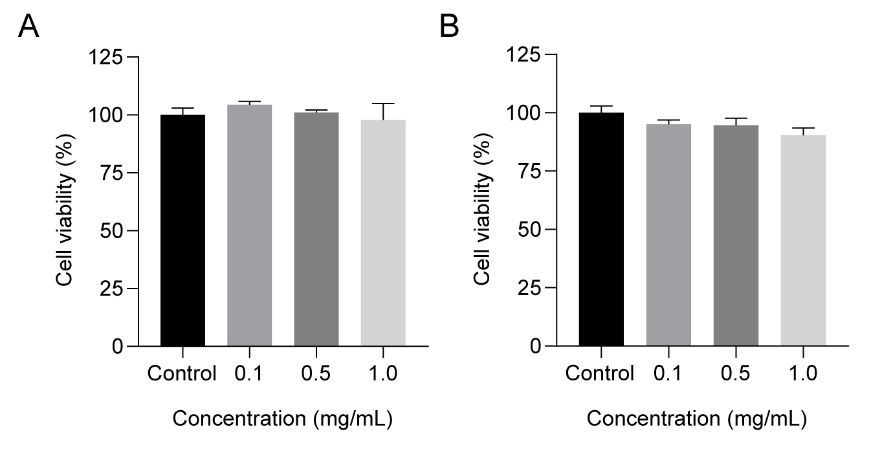


**Fig. S1.** Cell viability of Caco-2 cells treated with DPN (A) and SDPN (B). (Mean±SD, n=3)


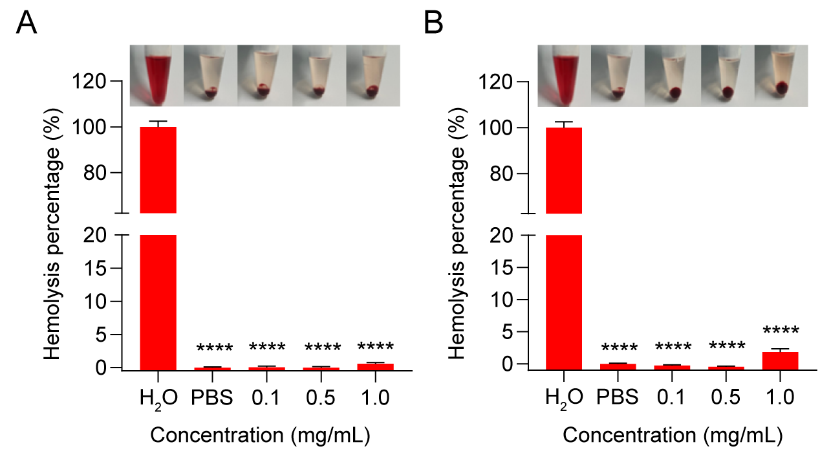


**Fig. S2**. Hemolysis percentage of DPN (A) and SDPN (B).


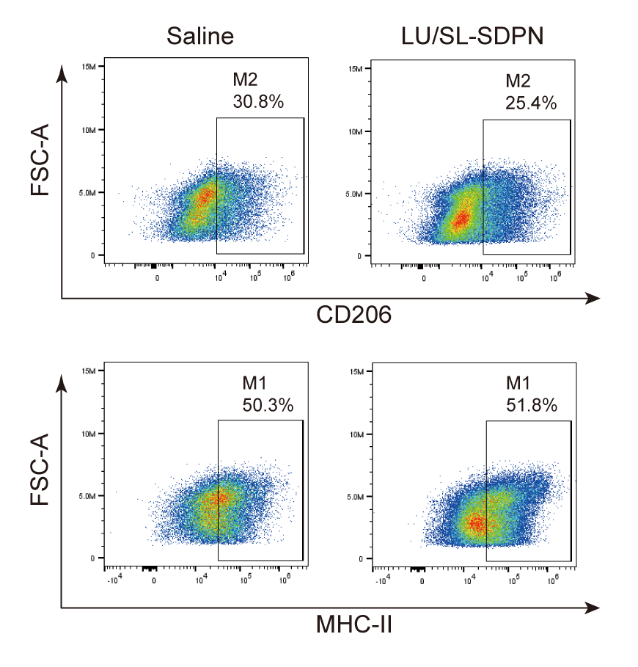


**Fig. S3.** Analysis of macrophages in 4T1 tumors by flow cytometry. Flow cytometric image of M2 (CD206^+^) and M1 (MHC-II^+^) proportion of saline and SDPN treated tumor tissues.


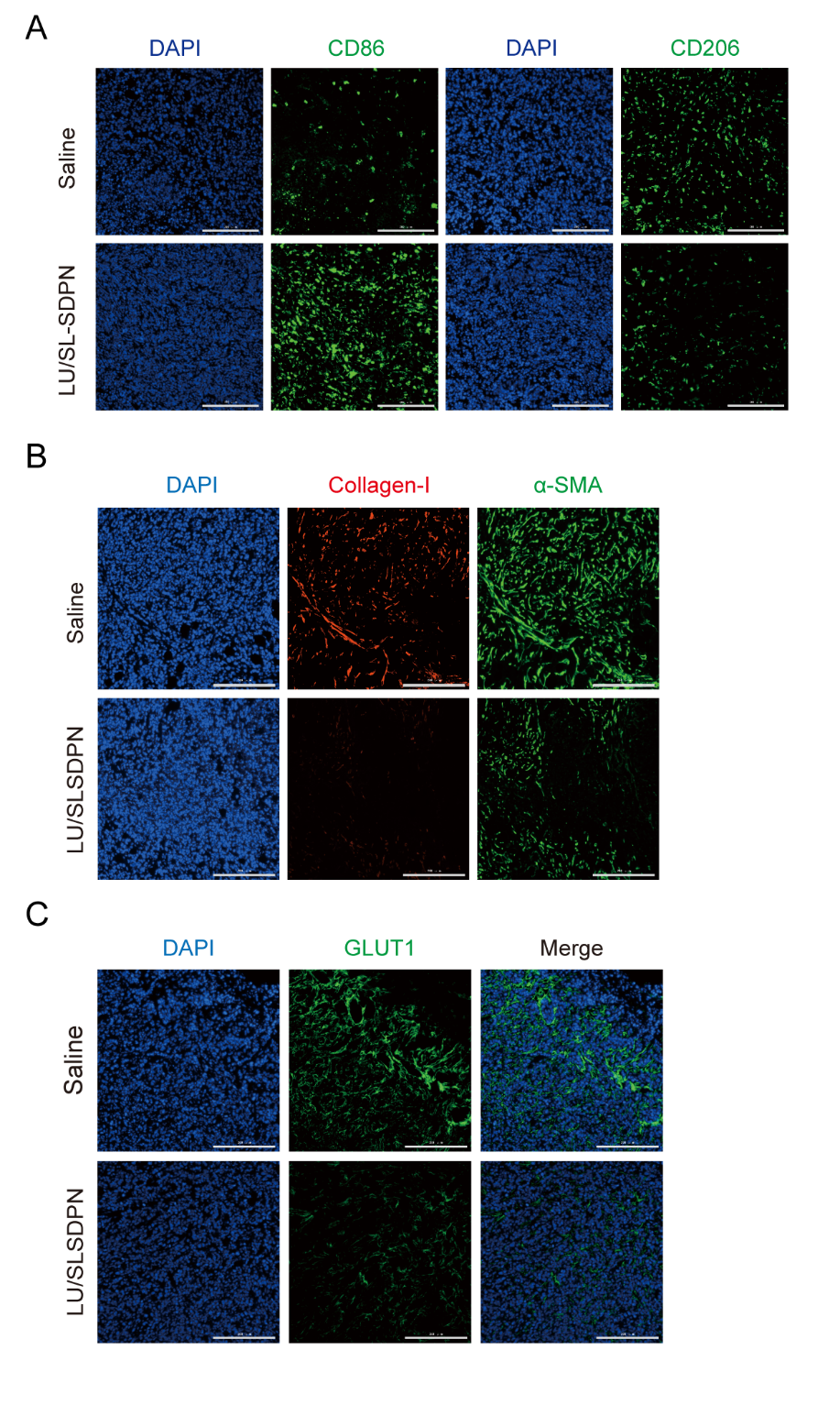


**Fig. S4.** (A) Immunofluorescence staining of tumor tissue sections for CD86 or CD206. (B) Immunofluorescence staining of tumor tissue sections for collagen I and α-SMA. (C)Immunofluorescence staining of tumor tissue sections for GLUT1.Scale bar=200 μm.


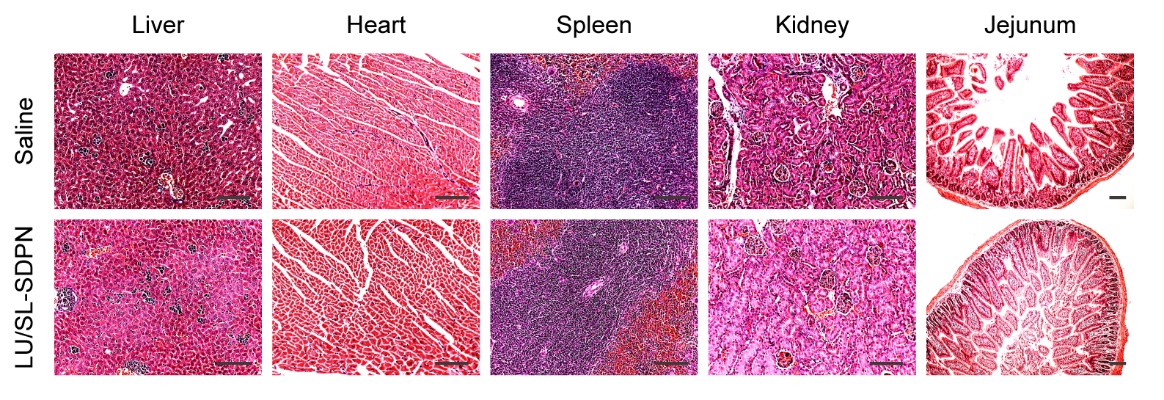


**Fig. S5**. Histological examination of the liver, heart, spleen, kidney, and jejunum in 4T1 tumor-bearing mice treated with saline and LU/SL-SDPN. Scale bar=100 μm.
